# The New Era FitScore test: a comprehensive general fitness assessment

**DOI:** 10.3389/fspor.2026.1764830

**Published:** 2026-05-13

**Authors:** Era Deva, Milaim Berisha, Pablo Prieto-González, Peter Sagat

**Affiliations:** 1 Faculty of Sport and Movement Science, University for Business and Technology (UBT), Prishtina, Kosovo; 2 Department of Physical Education, Global Sport Management, Seoul National University, Seoul, Republic of Korea; 3 Faculty of Sport and Movement Science, University for Business and Technology (UBT), Prishtina, Kosovo; 4 Sport Sciences and Diagnostics Research Group, GSD-HPE Department, Prince Sultan University, Riyadh, Saudi Arabia

**Keywords:** burpee, health, reliability, testing, validity, Witty SEM

## Abstract

**Background:**

This study developed a comprehensive fitness test, the New Era FitScore (NEFS), integrating full-body movement with reactive agility and neuromotor components.

**Methods:**

Ninety-six participants completed the NEFS: a 90-s circuit combining burpees with reactive sprints triggered by randomized Witty SEM lights. Reliability was assessed using Intraclass Correlation Coefficient (ICC) and Bland-Altman (bias, 95% limits), and predictors of NEFS performance were examined using linear regression.

**Results:**

Body composition showed significant negative correlations with NEFS across all groups. In female students, fat percentage demonstrated the strongest association (*r* = −0.631, *p* = 0.002), followed by BMI (*r* = −0.535, *p* = 0.010). Male students showed significant correlations with body mass (*r* = −0.578, *p* = 0.015) and BMI (*r* = −0.578, *p* = 0.015). In sedentary women, fat percentage again showed the highest correlation (*r* = −0.638, *p* < 0.001), and regression analysis revealed that fat percentage explained 39%–40% of NEFS variance across female groups. Among U15 soccer players, NEFS correlated strongly with Yo-Yo test distance and estimated VO₂max (*r* = 0.742, *p* = 0.002), while body composition showed weaker associations. Overall test-retest reliability was high [ICC (2,1) = 0.940, 95% CI:.906–0.961] and was broadly consistent across groups, with ICC values ranging from.812 to.973.

**Conclusion:**

Based on the study sample and analysis, the NEFS is a valid and reliable multidimensional assessment evaluating muscular strength-endurance, aerobic capacity, agility, and coordination in a single 90 s protocol. With favorable neuromuscular properties and minimal equipment needs, it is a practical tool for fitness evaluation in sports, health, and other communities.

## 1 Introduction

Performance testing is integral to sports science, providing a controlled environment to evaluate physical capacities, monitor progress, and inform individualized training and rehabilitation strategies (1, 2). It helps to identify physical strengths, limitations, and potential injury risks, thereby supporting the development of targeted and effective interventions (3, 4). Such assessments are essential for optimizing key performance attributes.

Beyond athletic performance, fitness evaluations are essential for promoting overall health, guiding rehabilitation, and supporting long-term well-being (5). They aid in identifying health risks, tracking recovery, and designing personalized exercise programs aligned with individual objectives (6). These assessments inform evidence-based practices in both clinical and community health contexts. Regular evaluations can encourage sustained physical activity, prevent chronic disease, and enhance quality of life across diverse populations (7).

There are over 400 distinct fitness tests, each targeting specific physical attributes (8). Fitness assessments have evolved into more specialized tests, often combining multiple tests to provide a comprehensive analysis of physical capabilities (9, 10). Standardized test batteries such as Eurofit (11), ALPHA-Fit (12), and the Fitness Map of Europe (13) offer structured protocols for evaluating various fitness components. Technological advancements have also led to the development of innovative assessment methods, enabling more precise, adaptable, and efficient evaluations that enhance progress monitoring and the personalization of training programs (14).

However, the growing complexity of fitness assessments makes test selection challenging, as it affects accuracy, relevance, and efficiency (10). Time constraints often require prioritizing certain tests, risking incomplete profiles. High costs of advanced technologies further limit accessibility, particularly for smaller organizations (15). Balancing comprehensiveness with practicality is essential, highlighting the need for efficient, cost-effective assessment methods.

Fitness is generally described as the state of being fit (16), and is often linked to the ability to perform daily activities. Physical fitness can be broadly classified into two categories: health-related fitness and skill-related fitness (17). Health-related fitness, also known as physiological fitness, reflects an individual's capacity to perform daily activities efficiently while reducing the risk of chronic disease (18). Its primary components include body composition, muscular strength, muscular endurance, cardiovascular endurance, and flexibility (19).

Skill-related fitness encompasses balance, coordination, agility, speed, power, and reaction time—attributes that extend beyond athletic performance to support functional movement, injury prevention, and overall physical competence across diverse populations (20). These neuromuscular qualities enhance movement efficiency and are increasingly recognized as essential components of comprehensive fitness assessment. Modern testing protocols should therefore integrate both health-related and skill-related dimensions to capture the full spectrum of physical function and provide actionable insights for training and rehabilitation.

Fitness tests can assess various aspects of physical fitness when conducted according to established principles (21). Test selection should reflect the sport's or assessment's goals, taking into account movement, physiology, and motor skills (22).

Traditional fitness assessments typically require multiple tests to measure various fitness components, which can be time-consuming and impractical in limited-time settings. Additionally, some tests lack relevance to real-world physical demands, reducing their practical applicability. Balancing specificity and practicality is key to obtaining useful results.

With that said, this paper focuses on designing a comprehensive fitness assessment protocol to evaluate an individual's overall physical fitness. This protocol will incorporate burpees as a central exercise, leveraging their full-body engagement and ability to assess multiple fitness attributes simultaneously.

The burpee, a full-body exercise developed by Royal Huddleston Burpee Sr. in 1939, was initially created to assess general fitness by measuring heart rate recovery (23). During World War II, the U.S. military adopted and intensified the test to evaluate recruits' endurance, agility, strength, coordination, and cardiovascular fitness (24). While primarily anaerobic, performing burpees continuously also offers aerobic conditioning, making it a practical and cost-effective fitness assessment.

Burpees have since been incorporated into various fitness tests, such as the McCloy Physical Fitness Test (25), and the 3-Minute Burpee Test (3MBT). The 3MBT is widely used to assess strength endurance, with various modifications developed by researchers to adapt the test for different populations and specific assessment goals. Previous research has shown correlations between burpee performance and cardiorespiratory fitness (26), with some adaptations made for specific populations, such as individuals with Down Syndrome (27).

Our 90 s test enhances traditional burpee assessments by integrating the Witty SEM Light system, which captures key performance metrics beyond repetitions. This technology measures reaction time, change of direction, and response to visual stimuli, adding cognitive and neuromuscular dimensions to the evaluation (28). By combining physical and perceptual demands, the protocol provides a more comprehensive, sport-relevant assessment of overall fitness and athletic performance.

This paper aims to design and validate a comprehensive fitness assessment protocol centered on burpees to evaluate overall physical condition. It addresses limitations of existing tests, such as time constraints, complexity, and narrow focus. By leveraging burpees' engagement of multiple muscle groups and energy systems, combined with the Witty SEM Light system to capture reaction time and movement agility, the test offers a holistic measure of strength, endurance, coordination, and reactive-motor skills within a practical 90 s timeframe. The protocol seeks to become a standardized, accessible, and efficient tool for research and applied use, adaptable to diverse populations without compromising accuracy or relevance. Ultimately, it contributes to developing more inclusive and practical fitness assessments for varied contexts.

## 2 Methods

### 2.1 Research design

This observational cross-sectional study with a test-retest design was conducted to develop and validate the New Era FitScore (NEFS) test. The study followed the COSMIN (COnsensus-based Standards for the selection of health Measurement INstruments) guidelines for assessing measurement properties of performance-based tests (29). Reporting adheres to the STROBE (Strengthening the Reporting of Observational Studies in Epidemiology) statement (30).

Table 1 presents our two-phase study model, where we evaluated three distinct groups: U15 male soccer players, male and female students, and sedentary adult women, using a combination of the New Era FitScore (NEFS) test and established validation measures. Phase 1 began with every participant performing the NEFS protocol to obtain a baseline FitScore. All groups then underwent body composition analysis via the Tanita scale; additionally, soccer players completed the Yo-Yo Intermittent Recovery Test Level 1 to estimate V˙O₂max and the 10/5 Repeated Jumps Test (MyJump2 app) to calculate Reactive strength (RSI). Phase 2, scheduled 7 days later, involved each participant repeating only the NEFS protocol. This two-step approach allowed us to assess validity by correlating initial FitScore results with body composition, aerobic endurance, and reactive strength measures, and determine the test-retest reliability of the FitScore by comparing Phase 1 and Phase 2 performances.

**Table 1:** Model of the study.

Sample	Phase 1 Testing	Phase 2 Testing (7 Days After)
Football Players	FitScore New Era Tanita YoYo Repeated Jumps	New Era FitScore
Students	FitScore New Era Tanita	New Era FitScore
Sedentary Women	FitScore New Era Tanita	New Era FitScore

### 2.2 Sample

The sample consisted of 96 individuals, divided into four groups: male soccer players (U15), male students, female students, and sedentary adult women, providing a heterogeneous population. The groups included 24 male soccer players (U15), 24 male students, 24 female students, and 24 women. The inclusion of adolescent athletes enabled assessment of the test's responsiveness to higher fitness levels and sport-specific conditioning, while the student groups represented moderately active adolescents. The adult women group, characterized by lower physical activity levels, enabled the evaluation of the test's sensitivity in detecting variations in general health-related fitness. This diversity in age, sex, and activity level enhances the generalizability of the findings and supports the test's potential applicability in both athletic and general populations. Participants over the age of 75 and under age of 35 were excluded to ensure physiological consistency. The heterogeneous sample (*n* = 96; ∼24 per group) supports ICC-based reliability analysis across diverse fitness levels (31).

Table 2 presents the mean (X¯) and standard deviation (±SD) of age, height, and body mass across four groups: soccer players, female students, male students, and adult women. The data highlights notable differences in demographics.

**Table 2:** Descriptive statistics for each group.

Groups	X¯ ±SD		
	Age	Height	Body Mass
Soccer Players	13.85 ± 0.75	161.5 ± 8.9	49.1 ± 9.8
Female Students	15.72 ± 0.79	164.5 ± 5.5	58.8 ± 8.4
Male Students	15.45 ± 0.69	160.6 ± 3.2	70.2 ± 18.4
Sedentary Women	41.78 ± 6.4	162.1 ± 5.8	69.5 ± 9.1

The study was conducted in accordance with the Declaration of Helsinki and approved by the Scientific Ethics Commission of the University for Business and Technology (UBT), approval no. 6806/45. All participants (or legal guardians for minors) provided written informed consent prior to participation.

### 2.3 Test protocol

**Figure 1:**
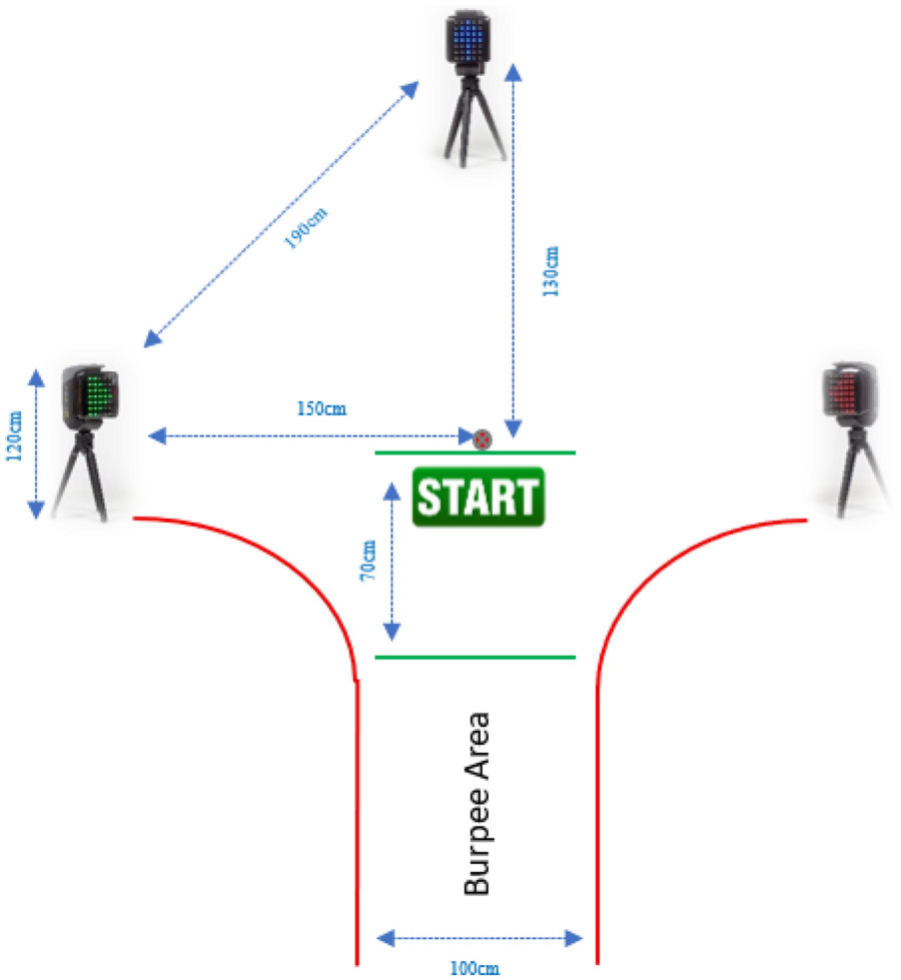
New Era FitScore test layout.

**Figure 2:**
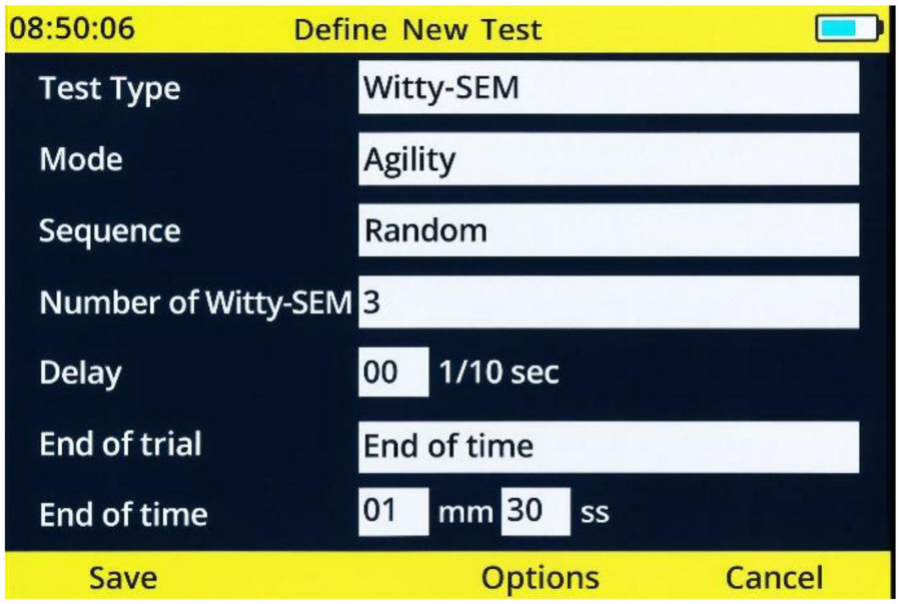
Test settings.

The test begins at the marked start line, where the participant waits for one of three Witty SEM lights to turn green in a randomized sequence. One light is positioned 130 cm in front of the start line, while the other two are placed 150 cm to the left and right, creating a 300 cm span between them. When the front light activates, the participant runs forward to touch it; when a side light activates, the participant runs laterally to reach it. After touching the activated light, the participant must return to the start line by running backwards and proceed to the designated burpee area to perform one full burpee (see Figure 1). This sequence, touching the light and completing a burpee, constitutes one lap. The goal is to complete as many laps as possible within 90 s (see Figure 2). The total number of completed laps reflects the individual's performance, with a higher number indicating better fitness. During the burpee, the athlete put the chest on the floor with the hands in a push-up position. When standing up, the athlete may rise onto both feet or directly into one leg forward, ready to run. In this position no jump is required. The athlete may look to find out which light is on while getting up. After bringing the hand close enough to deactivate the light, the athlete must walk or run back, noting that turning is not allowed.

Table 3 shows that the NEFS is a multidimensional test involving several movement phases, biomechanical and functional characteristics, and multiple energy systems. Overall, it suggests that NEFS assesses whole body performance, especially strength, coordination, agility, power, and fatigue resistance within a 90 second effort. A standardized warm-up consists of 5 min of free movements involving all body parts, followed by burpees, stepping forward and backward, and Witty SEM light “touch” drills that imitate the NEFS test movements.

**Table 3.** Multidimensional Classification of the New Era FitScore (NEFS) Test Components

Category	Classification	Motor Skill Sub-Category	Description	Source
Phases	1. Squat (Lower Body Flexion & Extension)	Strength, Flexibility, Mobility	Engages lower body	[32]
	2. Jump Back (Hip Hinge & Arm Support)	Agility, Stability, Core Strength, Mobility, Coordination	Engages core and upper body stability	[33]
	3. Plank/Push-Up (Horizontal Pressing)	Strength, Endurance, Stability	Upper body and core engagement	[34]
	4. Jump Forward (Knee & Hip Flexion)	Agility, Coordination, Explosiveness	Dynamic core and lower body movement	[33]
	5. Standing up (Triple Extension: Ankles, Knees, Hips)	Power, Speed, Coordination	Full-body power and explosive movement	[35]
	6. Run to Touch Witty SEM Lights (Front, right-left, backwards)	Speed, Acceleration, Reaction Time, Spatial orientation, Decision making	Sprint to touch the light and back to the burpee	[36, 37]
Biomechanical type	Multi-joint movement	Coordination, Strength	Involves ankles, knees, hips, shoulders, elbows	[26]
	Cyclic-acyclic movement	Coordination, Agility, Endurance	Repetitive but consists of distinct phases	[37]
	Ballistic movement	Power, Speed, Explosiveness	Includes explosive jumping and pushing actions	[39]
Functional classification	Total-body exercise	Strength, Endurance, Coordination	Engages core, upper and lower body simultaneously	[40]
	Closed kinetic chain (CKC), Opened kinetic chain (OKC)	Stability, Balance, Strength	Requires ground contact for force application	[41]
	Compound movement	Coordination, Strength, Mobility	Incorporates multiple movement patterns at the same time	[42]
Energy system involvement	Anaerobic-alactic	Power, Speed, Explosiveness	Short bursts, high power	[43]
	Anaerobic-lactic	Endurance, Strength	If performed for extended repetitions	
	Aerobic	Endurance	Continues low/moderate-intensity prolonged activities	
Test duration (90 secs)	Aerobic-anaerobic	Muscular Endurance, Fatigue Resistance	Time allocated for the test	[44]

The warm-up and the test are performed on the same surface, using a well-fixed mat on the floor. Both are conducted indoors, because the mat must be clean and dry to allow the participant to put the chest on the floor safely and consistently.

The test is applied only when the participant is fully recovered from previous training, with no heavy training in the last 72 h, if no specific protocol is being targeted (e.g., testing under fatigue).

To avoid the impact of the learning effect in test-retest, a familiarization session is implemented before testing.

Due to safety reasons, the NEFS test includes lying down and standing up, it is self-tempo and does not impose any requirement to move at maximal speed. Therefore, individuals who can independently lie down and stand up (i.e., perform basic bed-to-stand and walking movements) can safely participate using a mat for ground contact. A spotter is present throughout testing to supervise and ensure safety, and participants are permitted to stop the test at any time for any reason.

Test settings were configured for a Witty-SEM agility protocol with a random sequence, using three Witty-SEM units, 0.0 s delay, and a time-limited trial of 1 min 30 s (see Figure 2).

### 2.4 Validity

Validity refers to the extent to which a test accurately measures what it is intended to measure. It is a critical component of test evaluation, ensuring that the assessment provides meaningful and reliable data for fitness analysis (45, 46). To assess the validity of the FitScore test, several approaches were used, including content, construct, and concurrent validity. Content validity was ensured through consultations with sports science experts, who reviewed the test protocol to confirm that it accurately captured the targeted qualities and aligned with current performance testing standards. Specifically, the experts (5 sports scientists) were provided with the full protocol and test details, and they evaluated aspects such as test duration, content, light positioning, playing area, and procedural regulations, including failure criteria, after which their feedback was incorporated to refine the final version of the test. Regarding construct validity, convergent validation was performed to examine whether FitScore accurately reflected the overall level of fitness. Finally, the convergent validation was done by comparing FitScore results with participants' performance on well-known fitness indicators, such as V˙O₂max estimates from the Yo-Yo Intermittent Recovery Level 1 test and the Reactive Strength Index (RSI) from the 10/5 repeated vertical jumps. These comparisons allowed us to determine whether FitScore produces results similar to those of other valid tests administered on the same day.

The Yo-YoRL1 and RSI test were administered only to the soccer players because the primary criterion for athletes' fitness is performance capacity (endurance, maximal and reactive strength, etc.), where such tests are both more appropriate and practically meaningful. In contrast, in non-athlete populations, the health profile is typically described and monitored more through health-related indicators, especially body composition-based features, which are commonly used external criteria for this group. Therefore, performance tests were prioritized for athletes, while health parameters were prioritized for non-athletes, reflecting the different purpose and relevance of validation outcomes across populations.

#### 2.4.1 Tests used to assess validity

Tanita Scale: The Tanita Body Composition Scale uses Bioelectrical Impedance Analysis (BIA) to measure analyzed body features in this study such as W, BMI, and F% (47). It provides a detailed picture of body composition. In this study, FitScore results were compared to Tanita measurements to evaluate alignment with established body metrics.

Yo-Yo Intermittent Recovery Test Level 1 (YoYoRL1): This test evaluates an individual's ability to perform repeated high-intensity aerobic efforts. Participants run shuttles with timed beeps and 10-second recoveries until exhaustion. Total distance is used as a measure of endurance and aerobic capacity (47).

V˙O₂max (Estimated): V˙O₂max was estimated from YoYoRL1 results using the formula: V˙O₂max = (distance × 0.0084) + 36.4 (48). These estimates were compared to FitScore outcomes to assess how aerobic capacity relates to overall fitness performance.

Repeated Jumps Test (10/5): Using the MyJump3 App on an iPhone 13 (240 fps slow-motion recording), participants performed vertical jumps to assess reactive strength. The Reactive Strength Index (RSI) was calculated as jump height divided by contact time (49). All videos were analyzed by a trained assessor following a standardized procedure, and RSI scores were compared with FitScore results to examine the contribution of explosive power to fitness performance.

### 2.5 Reliability

Reliability refers to the degree of precision (consistency) when a test is repeated under similar conditions (50, 51). To assess the reliability of the New Era FitScore (NEFS) test, we used a test-retest design within a two-phase study. Participants first performed the NEFS protocol in Phase 1 to establish a baseline FitScore. Seven days later, in Phase 2, participants repeated the NEFS protocol under similar conditions. This design enabled us to evaluate test-retest reliability by comparing FitScore results across the two phases. The same examiner conducted both sessions to ensure consistency in test administration and scoring, supporting intra-rater reliability.

FitScore might assess: The New Era FitScore (NEFS) test is a multidimensional fitness assessment designed to evaluate a range of physical attributes within a single 90 s protocol. By combining reactive running tasks with repeated burpee execution, the test challenges participants' agility, coordination, and reaction time, as they respond to randomized visual stimuli and change direction quickly. Simultaneously, the repeated full-body effort of performing burpees under time constraints tests muscular and cardiovascular endurance.

Benefits: The proposed fitness assessment offers several key benefits, making it both practical and highly efficient. It captures multiple physical attributes, such as strength, endurance, coordination, and reaction time, in a single test. Additionally, the protocol is highly adaptable, with scalable intensity and customizable pacing, making it suitable for a wide range of populations, including athletes and sedentary individuals. The interactive and engaging nature of the test, particularly with the integration of FitLights, not only increases participant motivation but also encourages greater effort, leading to more accurate and meaningful results.

### 2.6 Statistical analysis

Data analysis for this study was conducted using the SPSS 27 statistical software package. The normality of the data was assessed using the Shapiro–Wilk test, which confirmed that the data followed a normal distribution (*p* > 0.05), allowing for the use of parametric statistical methods. We conducted a one-way ANOVA to determine whether there were statistically significant differences between the groups across all categories, as this was our aim. The results confirmed that all groups differed from each other (*p*-value = 0.005), indicating statistically significant differences. To assess the validity of the NEFS test, Pearson correlation (*α* = 0.05) analysis was conducted between NEFS scores and various health-related measures, including body composition and motoric features. The test-retest reliability of the NEFS was evaluated using the intraclass correlation coefficient (ICC) based on a two-way random-effects model, single measurement, absolute agreement [ICC (2,1)], comparing the two sets of scores. Also, Bland-Altman plots to evaluate test-retest agreement, reporting the mean bias and 95% limits of agreement between the two NEFS measurements.

To determine whether body composition and motor ability variables could predict NEFS test performance, linear regression was used. Due to high intercorrelations among predictors and multicollinearity, the backward elimination method was applied to refine the regression model. Finally, preliminary reference percentiles for each group were established using percentile statistics, categorizing performance into three levels based on the 25th, 50th, and 75th percentiles. Despite its preliminary nature, it provides available data that may offer useful reference information for readers.

## 3 Results

In female students, Table 4 indicates statistically significant correlations between the NEFS test and Fat% (*r* = −0.631, *p* = 0.002) and BMI (*r* = 0.535, *p* = 0.010), with BMI being influenced by other body composition features. However, body mass (*r* = −0.419, *p* = 0.052), which does not reflect the composition of body mass, was not significantly correlated with the NEFS test. Regarding male students, significant correlations between the NEFS test and body mass (*r* = −0.578, *p* = 0.015) and BMI (*r* = −0.578, *p* = 0.015) were observed, despite these components not reflecting the composition of mas or index. However, body fat percentage (*r* = −0.038, *p* = 0.885) was not significantly correlated with the NEFS test.

**Table 4:** Convergent validity through the correlations between body composition tests and NEFS score in male and female students.

Group	Variables	Correlation	NEFS1	NEFS2	NEFS (1_2)
♀	W	R	−0.461*	−0.358	−0.419
		P	0.020	0.102	0.052
	BMI	R	−0.504*	−0.450*	−0.535*
		P	0.010	0.036	0.010
	F%	R	−0.625**	−0.494*	−0.631**
		P	0.001	0.019	0.002
♂	W	R	−0.499*	−0.516*	−0.578*
		P	0.025	0.034	0.015
	BMI	R	−0.534*	−0.502*	−0.578*
		P	0.015	0.040	0.015
	F%	R	0.000	−0.054	−0.038
		P	0.999	0.837	0.885

* Correlation is significant at the 0.05 level.

** Correlation is significant at the 0.01 level.

Table 5 indicates statistically significant correlations between the NEFS test and body mass (*r* = −0.415, *p* = 0.031), BMI (*r* = −0.497, *p* = 0.008), and fat percentage (*r* = −0.638, *p* = 0.000), with BMI being influenced by other body composition features. These results support the validation of the NEFS test as a high-level health predictor.

**Table 5:** Convergent validity through the correlation of body composition with NEFS test in adult sedentary women.

Variables	Correlation	NEFS1	NEFS2	NEFS (1_2)
W	R	−0.402*	−0.416*	−0.415*
	P	0.038	0.031	0.031
BMI	R	−0.488**	−0.492**	−0.497**
	P	0.010	0.009	0.008
F%	R	−0.654**	−0.607**	−0.638**
	P	0.000	0.001	0.000

* Correlation is significant at the 0.05 level.

** Correlation is significant at the 0.01 level.

Table 6 indicates statistically significant correlations between the NEFS test and Fat% (*r* = −0.514, *p* = 0.050), YOYO test (*r* = 0.742, *p* = 0.002), and V˙O2max (*r* = 0.742, *p* = 0.002). Notably, RSI demonstrated a significant correlation with NEFS1 (*r* = 0.545, *p* = 0.036). These results support the validity of the NEFS test as a health predictor at a high level. However, two components, body mass (*r* = 0.042, *p* = 0.881) and body mass index (*r* = −0.030, *p* = 0.915), which do not provide information on the composition of body mass or index, were not significantly correlated with the NEFS test.

**Table 6:** Convergent validity through the correlation of body composition and physical fitness tests with NEFS score in U15 male soccer players.

Variables	Correlation	NEFS1	NEFS2	NEFS (1_2)
W	R	−0.060	0.105	0.042
	P	0.801	0.709	0.881
BMI	R	0.026	−0.053	−0.030
	P	0.913	0.852	0.915
F%	R	−0.421	−0.527*	−0.514*
	P	0.064	0.043	0.050
Yo-YoRL1 Distance	R	0.608**	0.721**	0.742**
	P	0.004	0.002	0.002
V˙O_2_max	R	0.608**	0.721**	0.742**
	P	0.004	0.002	0.002
RSI	R	0.545*	0.202	0.376
	P	0.036	0.489	0.185

* Correlation is significant at the 0.05 level (2-tailed).

** Correlation is significant at the 0.01 level (2-tailed).

Based on Table 7, the NEFS test demonstrated good to excellent test-retest reliability across all groups, with ICC values ranging from 0.812 in soccer players (*p* = 0.002) to 0.973 in sedentary women (*p* < 0.001). Female students (ICC = 0.860) and male students (ICC = 0.841) also showed high reliability, and the 95% confidence intervals for all groups further support the consistency and stability of NEFS scores over time. Based on Cohen's criteria, the observed effect sizes were negligible (ES < 0.20), aligning with the non-significant results and suggesting no significant pre–post differences in NEFS test scores.

**Table 7:** Reliability of NEFS test.

Groups	New Era Fit Score	Differences			Intraclass Correlation Coefficient (ICC)					Overall ICC		
		X¯ ±SD	Sig	ES	Intraclass Correlation^b^	Lower Bound*	Upper Bound*	Sig	Cronbach's Alpha	Sig	Lower Bound*	Upper Bound*
Female students	First test	15.68 ± 2.337	0.435	0.1	0.860	0.663	0.942	0.000	0.860	0.940	0.906	0.961
	Second test	15.95 ± 2.256										
Male students	First test	21.52 ± 3.223	0.783	0.1	0.841	0.562	0.943	0.000	0.841			
	Second test	21.35 ± 3.790										
Sedentary Women	First test	11.29 ± 1.877	0.001	0.2	0.973	0.941	0.988	0.000	0.973			
	Second test	11.92 ± 1.998										
Soccer players	First test	21.46 ± 1.846	0.030	0.1	0.812	0.441	0.937	0.002	0.812			
	Second test	22.53 ± 2.416										

* 95% Confidence interval.

**Figure 3:**
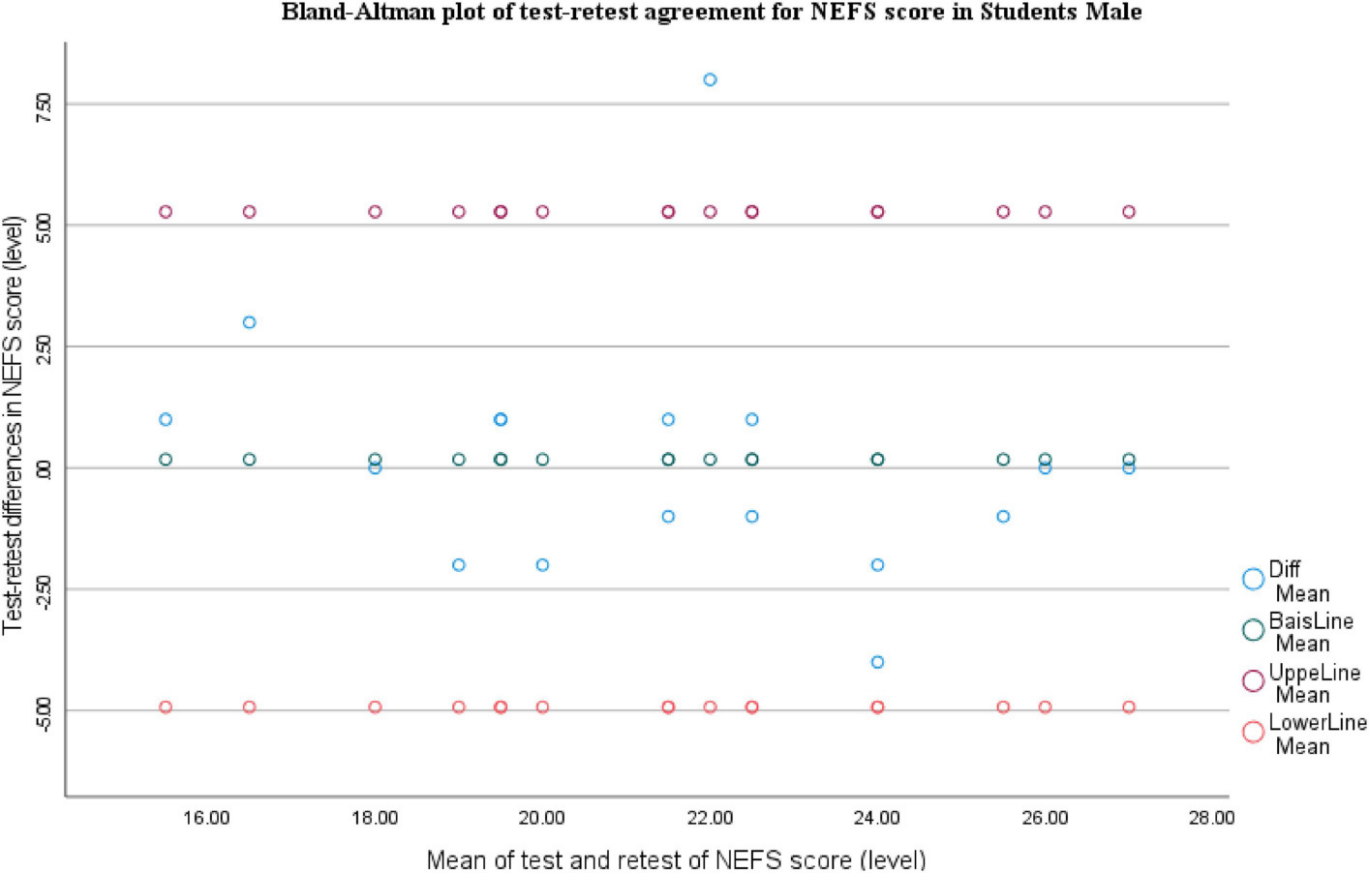
Bland-Altman plot of test-retest agreement for NEFS score in female students.

The Bland-Altman plot for NEFS shows very small bias (mean difference close to 0), with most points clustered around the zero line and almost all observations within the limits of agreement (∼−5 to +5). There's no clear trend across the measurement range, suggesting no proportional bias and good test-retest agreement overall (Figure 3).

**Figure 4:**
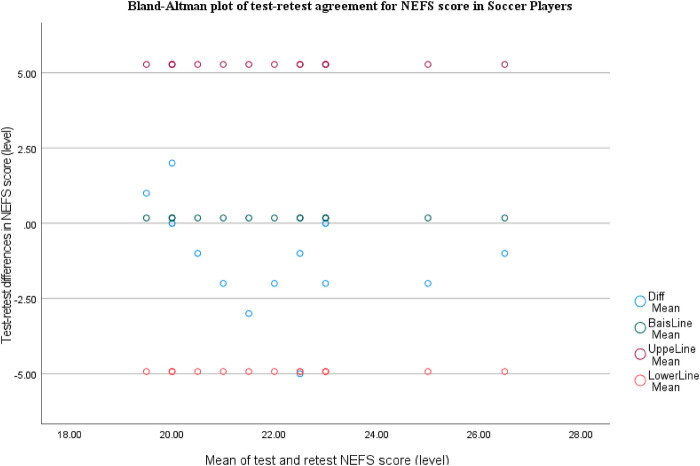
Bland-Altman plot of test-retest agreement for NEFS score in male students.

For NEFS, the Bland-Altman plot shows minimal bias (mean difference close to 0), with most points near the zero line and nearly all observations within the limits of agreement (∼−5 to +5). There's no obvious proportional bias across the score range, though there is one higher positive difference outlier, suggesting potential retest change (Figure 4).

**Figure 5:**
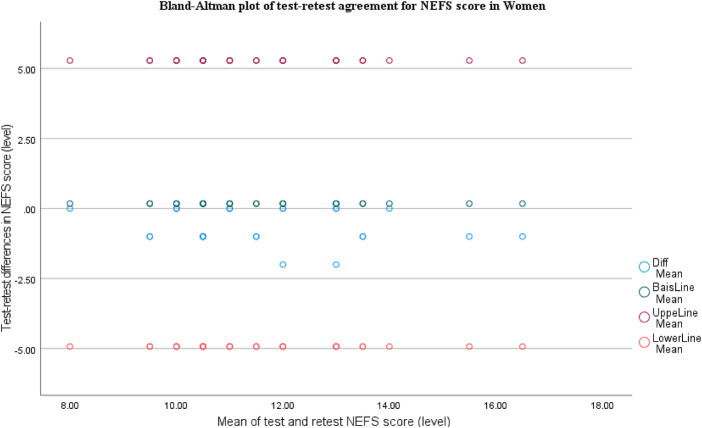
Bland-Altman plot of test-retest for NEFS score in women.

For NEFS, the Bland-Altman plot shows small bias close to zero (slightly negative), with most differences tightly clustered near the bias line and almost all points within the limits of agreement (∼−5 to +5). There's no clear proportional bias across the score range, indicating good test–retest agreement (Figure 5).

**Figure 6:**
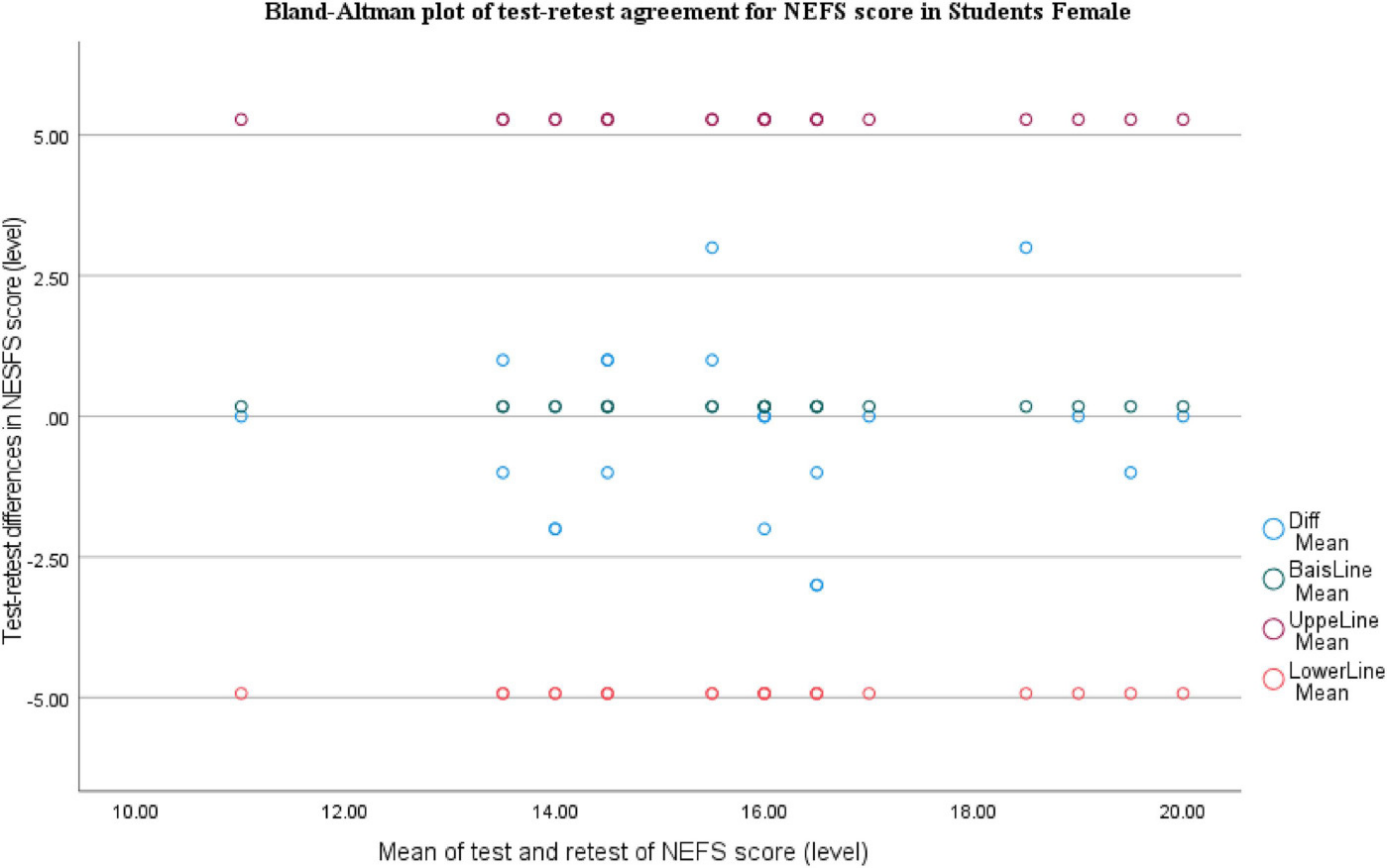
Bland-Altman plot of test-retest agreement for NEFS score in soccer players.

For NEFS, the Bland-Altman plot shows very small bias (mean difference close to 0, slightly negative), with most points near the bias line and almost all differences within the limits of agreement (∼−5 to +5). There's no clear proportional bias across the score range, although a few players show moderate negative retest differences, suggesting rare day-to-day variation (Figure 6).

Table 8 shows that in females, the model was significant (F = 13.262, *p* = 0.002), with each 1% increase in fat percentage linked to a 0.198 decrease in NEFS. It explained 39% of the variance (R² = 0.39), indicating a moderate fit. In males, the model was also significant (F = 7.529, *p* = 0.015); each 1 kg increase in body mass led to a 0.099 decrease in NEFS, explaining 33% of the variance (R² = 0.33).

**Table 8:** Explained variance in the NEFS test as the dependent variable by body composition features of students.

Gender	Model	Variables	F	Sig	Unstandardized Coefficient B	Standardized B	Sig.	R Square
♀	1	W (kg)	5.321	0.008	0.163	0.655	0.143	0.470
		BMI (kg/m^2^)			−0.238	−0.358	0.460	
		Fat (%)			−0.275	−0.876	0.033	
	2*	W (kg)	7.874	0.003	0.112	0.450	0.185	0.453
		Fat (%)			−0.319	−1.016	0.006	
	3*	Fat (%)	13.262	0.002	−0.198	−0.631	0.002	0.399
♂	1	W (kg)	2.802	0.082	−0.390	−2.276	0.289	0.393
		BMI (kg/m^2^)			0.862	1.563	0.438	
		Fat (%)			0.136	0.522	0.302	
	2**	W (kg)	3.985	0.043	−0.109	−0.639	0.014	0.362
		Fat (%)			0.047	0.180	0.441	
	3**	W (kg)	7.529	0.015	−0.099	−0.578	0.015	0.334

* Excluded variables: excluded in model 2 BMI (kg/m^2^); excluded in model 3 BMI (kg/m^2^); Body Mass (kg).

** Excluded variables: excluded in model 2 BMI (kg/m^2^); excluded in model 3 BMI (kg/m^2^); Fat Percentage (%).

The F-value (17.159) and significance level (*p* = 0.001) indicate that the model is statistically significant, demonstrating its effectiveness in predicting the dependent variable, NEFS. The unstandardized coefficient (B) for Fat Percentage reveals that a one-unit increase in body fat percentage is associated with a 0.247-unit decrease in NEFS score. According to Table 9, the model explains approximately 40% of the variance in NEFS (R² = 0.40), adjusted for the single predictor which is Fat Percentage (%). This indicates a moderate model fit, with a statistically significant contribution (*p* = 0.001).

**Table 9:** Explained variance in the NEFS test as the dependent variable by body composition features of women.

Model*	Variables	F	Sig	Unstandardized Coefficient B	Standardized B	Sig.	R Square
1	W (kg)	5.850	0.004	0.053	0.254	0.428	0.433
	BMI (kg/m^2^)			0.015	0.029	0.938	
	Fat (%)			−0.335	−0.866	0.012	
2	W (kg)	9.151	0.001	0.056	0.267	0.308	0.433
	Fat (%)			−0.330	−0.852	0.003	
3	Fat (%)	17.159	0.001	−0.247	−0.638	0.001	0.407

* Excluded variables: excluded in model 2 BMI (kg/m^2^); excluded in model 3 BMI (kg/m^2^); Fat Percentage (%).

Based on Table 10, the *F*-value for body composition (12.361, *p* = 0.001) shows the model significantly predicts NEFS. A one-unit increase in BMI raises the NEFS score by 0.687, while a one-unit increase in fat percentage lowers it by 0.588. The table shows that the model explains 67% of NEFS variance (R² = 0.67), with both predictors being significant (BMI: *p* = 0.002; Fat%: *p* = 0.001). For sports-specific tests, the F-value (15.776, *p* = 0.002) also indicates significance. A one-meter increase in Yo-Yo test performance raises NEFS by 0.006. The model accounts for 57% of the variance (R² = 0.57), reflecting a moderate fit (*p* = 0.002).

**Table 10:** Explained variance in the NEFS test as the dependent variable by tested features of soccer players.

Features	Model	Variables	F	Sig	Unstandardized Coefficient B	Standardized B	Sig.	R Square
Body composition	1	W (kg)	8.831	0.003	−0.066	−0.362	0.287	0.707
		BMI (kg/m^2^)			0.943	1.469	0.007	
		Fat (%)			−0.638	−1.490	0.001	
	2*	BMI (kg/m^2^)	12.361	0.001	0.687	1.069	0.002	0.673
		Fat (%)			−0.588	−1.372	0.001	
Sports specific test	1	YOYO Distance	7.710	0.008	0.006	0.708	0.006	0.584
		RSI (m/s)			1.270	0.133	0.533	
	2**	YOYO Distance	15.776	0.002	0.006	0.754	0.002	0.568

* Excluded variables: excluded in model 2 Body Mass (kg).

** Excluded variables V˙O_2_max was excluded from Model 1 due to high multicollinearity with YOYO Distance.; excluded in model 2 RSI (m/s); V̇O_2_max.

Table 11 presents the preliminary reference percentiles for each category, determined using the 25th, 50th, and 75th percentiles. While these preliminary reference percentiles are based on a limited number of participants and should be expanded in future studies, they provide a foundational reference for interpreting performance. These results offer an initial benchmark for distinguishing between good and less favorable outcomes on the NEFS test developed in this study. These values are exploratory, limited to the study sample, and not intended for population-level interpretation.

**Table 11:** Preliminary reference percentiles of each group category regarding NESFS test results.

Groups	X¯ ± SD	Percentiles		
		25th	50th	75th
Soccer player	22.0 ± 1.973	20.0	22.0	23.0
Female students	15.8 ± 2.152	14.3	16.0	16.6
Male students	21.4 ± 3.268	19.2	21.5	24.0
Sedentary Women	11.6 ± 1.913	10.5	11.0	13.0

## 4 Discussion

The primary goal of this study was to design a new, practical fitness assessment, the New Era FitScore (NEFS) test, intended to evaluate overall physical fitness in a time-efficient and comprehensive manner. To ensure the test could be deemed usable, its validity and reliability were subsequently evaluated across diverse populations.

The NEFS test was developed with the Burpee exercise as its foundation, chosen for its ability to engage multiple muscle groups while incorporating key components of fitness, within a single high-intensity bodyweight movement (52). The 90-second protocol integrated directional changes and reactive tasks using Witty SEM Lights to simulate real-life physical demands and evaluate participants' capacity to sustain high-intensity, full-body activity. The integration of the Witty SEM Lights system was a key advantage, as it introduced a cognitive (reacting to external stimuli) dimension to the test (29).

This test design is particularly relevant given the effects of aging on motor skills, including diminished control and coordination of physical movements (53). With age, muscle strength and coordination tend to decline, leading to slower reaction times, an ability influenced by both cognitive processing and physical responsiveness (54). Reaction time is further impaired under fatigue, as muscle exhaustion hampers responsiveness to central nervous system stimuli (55, 56). The NEFS test addresses these challenges by offering a time-efficient protocol that assesses not only reaction time under fatigued conditions but also evaluates cardiorespiratory fitness, muscular endurance, agility, coordination, and spatial awareness. By integrating cognitive (reacting to external stimuli) and physical demands, the NEFS test provides a more holistic measure of overall fitness than traditional assessments.

To determine the accuracy of the NEFS test, results were compared with well-established fitness indicators, including estimated V˙O₂max from the Yo-Yo Intermittent Recovery Test Level 1 (48), body composition from the Tanita scale (46), and reactive strength measured via the repeated jumps (10/5) test (49) using the MyJump3 app. This multi-metric approach aimed to validate whether the NEFS could serve as a reliable, accessible tool for assessing overall fitness. Importantly, each of these measures' maps onto categories of physical fitness: estimated V˙O₂max captures metabolic fitness (health-related) by reflecting the muscle's capacity for aerobic energy production relative to maximal oxygen uptake (57). Body composition analysis via the Tanita scale addresses health-related fitness (18, 19) while the repeated jumps test assesses skill-related fitness, namely reactive strength, coordination, and power elements crucial for movement efficiency and performance (20). This alignment ensures that the NEFS test not only integrates multiple movement patterns in its protocol but also comprehensively reflects health and skill dimensions of physical fitness. The tests were administered across a diverse population representing various ages, fitness levels, and athletic backgrounds. This diversity allowed for an evaluation of the test's applicability and consistency across different population groups.

The test demonstrated strong validity, as NEFS scores closely aligned with established indicators of physical fitness, particularly body composition (notably body fat percentage) and aerobic capacity, as reflected in V˙O₂max and Yo-Yo test performance. The correlation analyses conducted provide further evidence of the NEFS test's validity across various populations. Among female students, statistically significant negative correlations were found between NEFS scores and both fat percentage and BMI, indicating that as body fat and BMI increase, NEFS performance tends to decrease (see Table 4). This suggests that the NEFS test is sensitive to health-related fitness components, particularly in identifying the impact of body composition on overall functional performance. Similar patterns were observed among male students, with NEFS scores negatively correlated with both body mass and BMI, reinforcing the test's ability to reflect physical condition across genders. In sedentary women, the correlations were even more pronounced: significant negative correlations were found between NEFS and body mass, BMI, and fat percentage (see Table 5). These findings suggest that the NEFS test can effectively distinguish fitness levels even among less active populations, highlighting its potential as a screening tool for physical health and risk factors associated with sedentary behavior. These findings also support well-documented evidence that increases in body fat, body mass, and BMI negatively impact overall fitness performance (58, 59).

On the other hand, athletic populations also demonstrated meaningful results. In football players, NEFS scores showed a strong negative correlation with fat percentage, and a strong positive correlation with both the Yo-Yo test and estimated V˙O₂max (see Table 6). This reinforces the test's relevance in measuring aerobic fitness and body composition in performance-driven groups. Additionally, the Reactive Strength Index (RSI), a key measure of neuromuscular efficiency (60), showed a significant correlation with NEFS1 scores, suggesting a potential relationship between the test and power- and agility-related components of skill fitness.

Repeated testing in selected groups confirmed the test's reliability. NEFS scores were significantly higher on the second administration for both soccer players and women, suggesting improved performance potentially due to familiarization or training adaptation (61). Importantly, strong test-retest reliability was demonstrated by high correlation coefficients, indicating consistency of the NEFS across populations. Similarly, the absence of significant differences between female and male students, combined with strong NEFS1-NEFS2 correlations for both groups, further supports the test's reliability and gender-neutral applicability (see Table 7).

Our analyses demonstrated that body composition and performance variables significantly predicted NEFS scores across subgroups. In female students, fat percentage was a significant negative predictor of NEFS, with each 1% increase associated with a decrease of 0.198 in NEFS scores, explaining up to 39% of the variance. In male students, increased body body mass predicted lower NEFS scores (see Table 8).

The regression model for women was statistically significant, with fat percentage emerging as a strong negative predictor of NEFS scores, explaining 40% of the variance and indicating a moderate model fit (see Table 9).

Among soccer players, both BMI and fat percentage were significant predictors: higher BMI was associated with higher NEFS scores, and higher fat percentage was associated with lower scores, together explaining 67% of the variance. In athletic groups such as soccer players, a higher BMI typically indicates greater lean muscle mass rather than excess fat, with greater muscle contributing to enhanced strength, power, and overall fitness, thereby improving NEFS scores. Additionally, Yo-Yo test performance was a significant predictor, with each additional meter increasing NEFS by 0.006, accounting for 57% of the variance (see Table 10). These findings align with established principles in exercise science, reinforcing that higher aerobic capacity (62) and lower body fat (56) are strongly associated with better overall physical fitness, thereby supporting the NEFS test's validity in reflecting well-known fitness determinants.

Taken together, these findings provide support for the NEFS test as a valid and reliable multidimensional assessment of physical fitness. It captures both health- and skill-related fitness attributes, accommodates diverse populations, and responds to changes over time, establishing its utility in both athletic and general health contexts.

Our study indicates that the test tends to be anaerobically dominant when performed by high-performance populations, such as football players, due to their ability to sustain high-intensity efforts throughout the test. In contrast, for individuals with lower fitness levels, the test shifts toward a more aerobic emphasis, as they are unable to maintain the same intensity and instead rely on a more sustained, moderate effort (63). The cognitive (reactive agility, reacting to an external stimuli) component of the test becomes more prominent as participants perform burpees faster. Additionally, the movement mechanics observed during the test vary depending on the participant's fitness level and motor capabilities. This adaptability in movement execution allows the test to be easily performed by individuals across a wide range of fitness levels, making it accessible and inclusive for both athletic and general populations.

While this study offers valuable insights into the NEFS test's effectiveness, a few things should be kept in mind. First, the sample sizes for each group (ranging from 24 to 28 participants) were relatively small, so larger groups in future studies could help provide more confidence in the results. However, the NEFS test still showed consistent patterns across the different populations tested. Second, since the NEFS provides an overall assessment of physical fitness, it does not allow us to identify the specific strengths and weaknesses of the individuals tested. For our next step, we aim to break down the fitness components to better identify individual strengths and weaknesses.

## 5 Conclusion

The New Era FitScore (NEFS) test was developed as a practical, time-efficient, and comprehensive tool to evaluate overall physical fitness. By integrating reactive movements, directional changes, and full-body tasks such as the burpee, this 90 s protocol assesses multiple fitness domains, including strength, endurance, coordination, agility, and cognitive (reactive agility, reacting to an external stimuli) responsiveness. The findings of this study support the NEFS as a valid and reliable assessment across diverse populations, demonstrating strong correlations with established fitness indicators such as V˙O₂max and body composition, and moderate associations with reactive strength. Its versatility, simplicity, and engaging design make it suitable for a wide range of settings, from sports performance environments to clinical and general fitness contexts. While further research with larger, more heterogeneous samples is needed, the present evidence positions the NEFS as a promising, holistic, and accessible approach to modern fitness assessment.

## Data Availability

The raw data supporting the conclusions of this article will be made available by the authors, without undue reservation.
